# An evaluation of harvest plots to display results of meta-analyses in overviews of reviews: a cross-sectional study

**DOI:** 10.1186/s12874-015-0084-0

**Published:** 2015-10-26

**Authors:** Katelynn Crick, Aireen Wingert, Katrina Williams, Ricardo M. Fernandes, Denise Thomson, Lisa Hartling

**Affiliations:** School of Public Health, University of Alberta, Edmonton, Canada; Cochrane Child Health, The Cochrane Collaboration, Edmonton, Canada; Alberta Research Centre for Health Evidence, Department of Pediatrics, University of Alberta, 4-472 Edmonton Clinic Health Academy, 11405-87 Avenue, Edmonton, Alberta T6G 1C9 Canada; Department of Paediatrics, University of Melbourne, Developmental Medicine, Royal Children’s Hospital, Melbourne and Murdoch Childrens Research Institute, Edmonton, Canada; Department of Pediatrics, Santa Maria Hospital, Lisbon Academic Medical Centre and Clinical Pharmacology Unit, Instituto de Medicina Molecular, Faculty of Medicine, University of Lisbon, Lisbon, Portugal

**Keywords:** Meta-analysis, Systematic reviews, Overviews of reviews, Knowledge synthesis, Graphs, Data presentation, Harvest plots

## Abstract

**Background:**

Harvest plots are used to graphically display evidence from complex and diverse studies or results. Overviews of reviews bring together evidence from two or more systematic reviews. Our objective was to determine the feasibility of using harvest plots to depict complex results of overviews of reviews.

**Methods:**

We conducted a survey of 279 members of Cochrane Child Health to determine their preferences for graphical display of data, and their understanding of data presented in the form of harvest plots. Preferences were rated on a scale of 0–100 (100 most preferred) and tabulated using descriptive statistics. Knowledge and accuracy were assessed by tabulating the number of correctly answered questions for harvest plots and traditional data summary tables; t-tests were used to compare responses between formats.

**Results:**

53 individuals from 7 countries completed the survey (19 %): 60 % were females; the majority had an MD (38 %), PhD (47 %), or equivalent. Respondents had published a median of 3 systematic reviews (inter-quartile range 1 to 8). There were few differences between harvest plots and tables in terms of being: well-suited to summarize and display results from meta-analysis (52 vs. 56); easy to understand (53 vs. 51); and, intuitive (49 vs. 44). Harvest plots were considered more aesthetically pleasing (56 vs. 44, *p* = 0.03). 40 % felt the harvest plots could be used in conjunction with tables to display results from meta-analyses; additionally, 45 % felt the harvest plots could be used with some improvement. There was no statistically significant difference in percentage of knowledge questions answered correctly for harvest plots compared with tables. When considering both types of data display, 21 % of knowledge questions were answered incorrectly.

**Conclusions:**

Neither harvest plots nor standard summary tables were ranked highly in terms of being easy to understand or intuitive, reflecting that neither format is ideal to summarize the results of meta-analyses in overviews of reviews. Responses to knowledge questions showed some misinterpretation of results of meta-analyses. Reviewers should ensure that messages are clearly articulated and summarized in the text to avoid misinterpretation.

**Electronic supplementary material:**

The online version of this article (doi:10.1186/s12874-015-0084-0) contains supplementary material, which is available to authorized users.

## Background

Systematic reviews respond to the challenge of knowledge management by identifying, appraising, and synthesizing research evidence in an accessible format [1]. Knowledge management is a commonly cited barrier to knowledge translation and includes the volume of research evidence, time to read evidence, and the skills to appraise and understand research evidence [2]. Meta-analysis is used in systematic reviews to statistically combine quantitative results for the same outcome from two or more separate studies [3]. It permits the calculation of a single estimate and confidence interval of effect that integrates all the available information from the results of similar studies (e.g., all studies examining a specific intervention) [4].

Overviews of reviews are a relatively new form of knowledge synthesis that aims to bring together evidence from two or more systematic reviews, for example multiple systematic reviews examining different interventions for a single condition [5]. Overviews are considered a friendly front-end to systematic reviews, as they provide a single source of information regarding alternative treatment options for decision-makers [5]. Because overviews bring together multiple systematic reviews, they may contain a large volume of results and statistical measures.

Cochrane Child Health has been producing overviews of reviews since 2006 for *Evidence-based Child Health: A Cochrane Review Journal* [6]. To date over 30 overviews have been published in the journal. Typically, results from the individual systematic reviews are presented in detailed tables that provide, for each outcome and comparison, the number of studies, number of patients, effect estimates (e.g., summary estimate and confidence interval), a measure of statistical heterogeneity across studies (e.g., I^2^ statistic), numbers needed to treat or harm (as applicable), and sometimes an indication of the quality of evidence. Graphs may be useful in this context to assist with interpretation of data due to the volume and complexity of information [7].

It has been noted that “graphs are essential for effective communication in science;” [7] however, accepted data displays in health care research have been adopted largely on the basis of tradition rather than on the research of presentation methods [8]. An area that has received little attention in research is that of making data more meaningful and reducing the mental computational load of visual displays [9, 10]. Harvest plots are a novel method for graphically displaying evidence from complex and diverse studies or results, or effects of heterogeneous interventions [11]. Harvest plots were originally developed by Ogilvie et al. in the course of a systematic review to combine the graphical directness of a forest plot with a narrative account of what could be learned from a diverse group of studies [11]. The harvest plot approach to graphical displays developed by Ogilvie et al. has the potential for other uses, including presentation of data from overviews of reviews. In the context of overviews of systematic reviews, the harvest plot method is flexible in that quantitative data for all studies can be displayed when it would not be possible to combine in a traditional forest plot [12]. Moreover, harvest plots may be useful for results where outcomes are not identical, when study designs preclude them from being combined, or when data is reported in different formats [11, 12].

As part of our goal to advocate for decision-making based on finding, understanding and using the best available evidence, Cochrane Child Health aims to develop appropriate methods for displaying the results from knowledge synthesis and meta-analysis in child health. To this end, we conducted a survey to examine whether harvest plots can be adapted and applied as a means of synthesizing and reporting findings of systematic reviews in overviews of reviews. When considering the impact of data displays, three domains have been identified in the literature [9]: comprehension (or interpretation of the data); the way in which the display affects hypothetical choice or behavior in practice; and preference (or liking) for one display over another. The objectives of this study were to: 1) determine the feasibility of using harvest plots to depict complex results of overviews of reviews; 2) survey end users to determine their preferences for graphical display of data, and their understanding of data presented in the form of harvest plots; and 3) compare end users’ preferences and understanding of data displayed in the form of traditional tables alone to that of harvest plots used in conjunction with traditional tables in overviews of reviews.

## Methods

### Design and participants

This was a cross-sectional, randomized, descriptive study using an online survey. The survey was sent to 279 members of Cochrane Child Health which includes pediatric healthcare providers and researchers from around the world; this represents all members except for those involved in the design and conduct of this study. An initial email was sent to the member mailing list outlining the survey and requesting participation, along with a link to the electronic survey which was administered using REDCap software [13]. Using a modified Dillman approach [14], two reminder emails were sent in two-week intervals following the initial contact; the survey was closed after 6 weeks. The survey took approximately 10 min to complete. Participation was voluntary. As an incentive to participate, respondents had the opportunity to enter their name into a draw for an iPad. The study was approved by the University of Alberta Ethics Review Board prior to implementation of the survey.

### Data displays

**Fig. 1:**
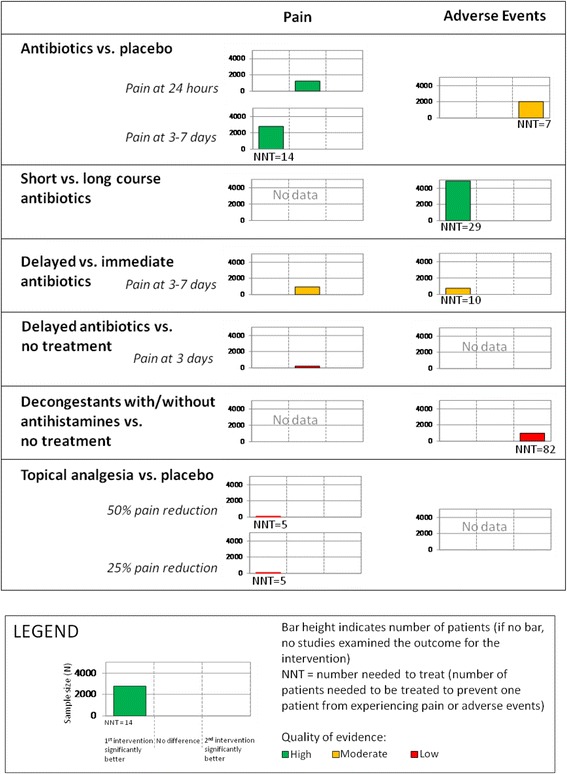
Harvest plots for overview of reviews on acute otitis media

**Fig. 2:**
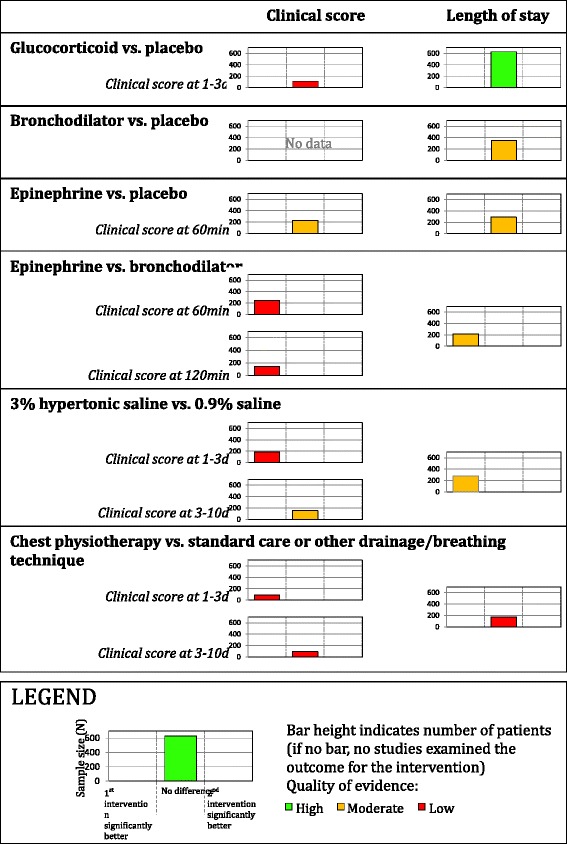
Harvest plots for overview of reviews on bronchiolitis

We developed harvest plots for two overviews of reviews that we had previously prepared and published in *Evidence-based Child Health* [15, 16]. The harvest plots for the two chosen overviews displayed six intervention comparisons for two outcomes (Figs. 1 and 2). We selected only two outcomes from each of the overviews in order to limit the length of the survey and optimize completion rates. Each row of the plot represented the outcomes for the specified comparison. Each plot contained a bar representing the number of participants contributing data for the outcome of interest for the specified comparison. Each vertical bar of the plot was colored to indicate the quality of evidence (based on the Grading of Recommendations Assessment, Development, and Evaluation (GRADE) criteria; www.gradeworkinggroup.org) for each comparison and outcome. A green bar indicated high quality of evidence, yellow indicated moderate quality, and red indicated low quality. There were no outcomes graded as very low; where no data were available, no grading was presented. Numbers needed to treat (NNT) were also included in the harvest plots where results were statistically significant. Prior to survey implementation, 6 individuals with experience in knowledge synthesis were invited to review the survey instrument for flow and understanding. An iterative process was undertaken for revisions and further reviews.

The tables for the two chosen overviews displayed the same six intervention comparisons and two outcomes. These tables were modified versions of those presented in the original overviews of reviews (see Table 1 for example); modifications included simplification due to selection of fewer outcomes, as well as slight edits to font, spacing, and color for visual appeal. The tables included the number of participants contributing data for each outcome, the effect estimate and 95 % confidence interval (CI), the number needed to treat (NNT) (where the effect was significant), I-squared statistic, and the quality of evidence (high, moderate, or low).

**Table 1:** Sample summary table presented in survey showing select results from overview of reviews on bronchiolitis

Bronchiolitis - inpatient outcomes								
Clinical score					Length of stay			
Time point for clinical score assessment	Patients (studies)	Effect estimate SMD (95 % CI)	I^2^ (%)	Quality of evidence	Patients (studies)	Effect estimate MD (95 % CI)	I^2^ (%)	Quality of evidence
**Glucocorticoids vs. placebo**								
At 1–3 days	113 (4)	−0.74 (−1.48,1.01)	70	Low	633 (8)	−0.18 (−0.39,0.04)	16	High
**Bronchodilator vs. placebo**								
No data					349 (6)	0.06 (−0.27,0.39)	0	Moderate
**Epinephrine vs. placebo**								
At 60 min	232 (2)	−0.04 (−0.49, 0.40)	46	Moderate	292 (2)	−0.35 (−0.87,0.17)	0	Moderate
**Epinephrine vs. bronchodilator**								
** At 60 min**	**248 (4)**	**−0.79 (−1.45,-0.13)** ^**a**^	**79**	**Low**	**261 (4)**	**−0.28 (−0.46,-0.09)** ^**a**^	**0**	**Moderate**
** At 120 min**	**140 (1)**	**−0.52 (−0.68,-0.18)** ^**a**^	**NA**	**Low**				
**3 % hypertonic saline vs. 0.9 % saline**								
** At 1–3 days**	**183 (3)**	**−0.84 (−1.39,-0.30)**	**66**	**Low**	**282 (4)**	**−1.16 (−1.55,-0.77)** ^**a**^	**0**	**Moderate**
At 3–10 days	156 (3)	−1.08 (−2.47,0.31)	93	Moderate				
**Chest physiotherapy vs. standard care or other drainage/breathing technique**								
** At 1–3 days**	**87 (1)**	**−0.55 (−0.98,-0.12)** ^**a**^	**NA**	**Low**	172 (3)	0.07 (−0.58,0.73)	0	Low
At 3–10 days	91 (2)	−0.14 (−0.81,0.53)	59	Low				

^a^favours 1st intervention; NA - not applicable; SMD - standardized mean difference

Outcomes in **bold** indicate statistical significance

Before viewing each display, participants were provided with a brief explanation of how to read and interpret the display. Three examples of properly interpreted results from each of the displays were provided. Participants viewed and responded to the knowledge questions for each display separately and in succession. Participants were asked a series of questions to test their knowledge and understanding of each of the displays. Participants were additionally asked if they had seen each of the display types before and their preference of each display. Participants were asked using a 100-point Likert scale if they felt each of the display types, when used alone, was well suited to summarize and display the results from meta-analyses, whether the display was aesthetically pleasing, easy to understand, and intuitive.

### Control of bias

**Fig. 3:**
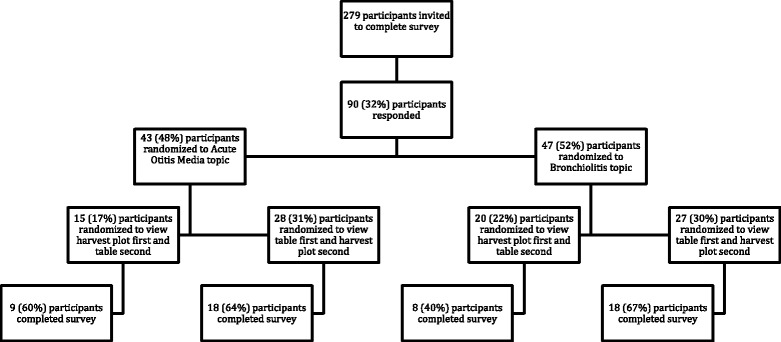
Participant recruitment and randomization

Participants were randomized to receive one of four surveys (Fig. 3). Participants were randomized first to one of the two overview topics (either acute otitis media or bronchiolitis) to control for the effects of context and framing on decision-making [17]. Within each topic, the order in which participants viewed the two display types was also randomized (i.e., participants viewed either the harvest plot or table first to minimize bias due to the learning effect).

### Statistical analysis

The analysis was divided into three parts: 1) knowledge assessment and accuracy; 2) preference; and 3) demographic characteristics (as well as experience with systematic reviews and related methods).

Knowledge and accuracy was assessed by tabulating the number of correctly answered knowledge questions for each type of display. Tabulations were done for each survey individually, as well as overall. Paired t-tests were used to assess differences in the number of correct answers within the acute otitis media and bronchiolitis topics comparing the harvest plot to the table.

Preferences and demographic characteristics were tabulated using descriptive statistics. Differences regarding preference for the harvest plot and table displays were tested using paired t-tests. We tested whether the groups of participants receiving the four different surveys differed with respect to demographic characteristics using ANOVA, grouping on topic and order of display.

## Results

Out of the 279 participants that were invited to complete the survey, 90 (32 %) participants’ responded. 37 (13 %) participants started, but did not complete, the survey and fifty-three individuals (19 %) completed the survey (Fig. 3; Table 2). The majority of respondents were female (60.4 %). Over half of respondents held a PhD (47.2 %), MD (37.7 %), or equivalent. Participants were from 7 countries; the majority were from the United States (*n* = 11; 20.8 %), Canada (*n* = 9; 17.0 %), UK and Ireland (*n* = 9; 17.0 %), and Australia (*n* = 8; 15.1 %). Demographic characteristics were not found to significantly differ by topic or order of display.

**Table 2:** Characteristics of respondents (*n* = 53)

Demographic characteristics		N	%
Gender			
	Female	32	60.4
	Male	21	39.6
Academic degrees			
	BA/BSc or equivalent	1	1.9
	MA/MSc or equivalent	4	7.6
	MD or equivalent	20	37.7
	PhD or equivalent	25	47.2
	Other	3	5.7
Country of academic affiliation			
	USA	11	20.8
	Canada	9	17.0
	UK/Ireland	9	17.0
	Australia	8	15.1
	Other	16	30.2
Experience with systematic reviews and meta-analysis		Median	IQR
	Number of systematic reviews published (per participant)	3	(1, 8)
	Number of systematic reviews published that contain at least one meta-analysis (per participant)	2	(1, 5)
	Number of journal articles published on the development of methods for systematic reviews (per participant)	0	(0, 0)
	Number of journal articles published specifically on development of meta-analysis methods (per participant)	0	(0, 0)

Respondents had published a median of 3 (inter-quartile range [IQR]: 1, 8) systematic reviews and 2 (IQR: 1, 5) systematic reviews containing at least one meta-analysis. Further, respondents had published: journal articles specifically on the development of systematic review methods (*n* = 13); journal articles on the development of meta-analysis methods (*n* = 4); or, other texts relevant to meta-analyses (e.g., book chapters, letters, editorials) (*n* = 22).

Only 7.6 % of respondents had seen a harvest plot before completing this survey while 52.8 % had seen a similar table to the one presented in the survey (Table 1). On a scale from 0 to 100 (where 100 was most favorable), average responses showed little difference between harvest plots and standard tables with respect to the following features (Table 3): well suited to summarize and graphically display the results from meta-analysis (mean 51.6 [standard deviation (SD) 26.9] harvest plots; 55.6 [SD 24.8] tables; *p* = 0.36); easy to understand (52.7 [SD 26.7] harvest plots, 50.7 [SD 26.2] tables; *p* = 0.70); intuitive format (48.8 [SD 25.6] harvest plots; 43.8 [SD 24.2] tables; *p* = 0.35). Respondents rated harvest plots as more aesthetically pleasing (56.3 [SD 29.0] harvest plots, 44.1 [SD 25.0] tables; *p* = 0.03).

**Table 3:** Preferences of respondents for harvest plot and table formats

	Harvest Plot		Table		
Variable (rated on a 100-point Likert scale)	Mean	(SD)	Mean	(SD)	p-value
This type of display is well suited to summarize and graphically display results from meta-analysis	51.6	(26.9)	55.6	(24.8)	0.36
This type of display is aesthetically pleasing	56.3	(29.0)	44.1	(25.0)	0.03
This type of display is easy to understand	52.7	(26.7)	50.7	(26.2)	0.70
This type of display is intuitive	48.8	(25.6)	43.8	(24.2)	0.35

On a scale of 0 to 100 (where 100 was most favorable), respondents were neutral on average (56.5 [SD 29.7]) as to whether the harvest plots were helpful in summarizing data from systematic reviews *in addition* to the tables. When asked if harvest plots could be used in conjunction with standard tables to display the results from systematic reviews, 39.6 % responded yes, 45.3 % responded yes if the harvest plots were improved, and 15.1 % responded no.

With respect to the series of knowledge questions, there was little difference in the number of correctly answered questions between the harvest plot and table displays for each topic. Out of 12 knowledge questions for the acute otitis media topic, 28.9 % of the questions were answered incorrectly for the harvest plot and 23.2 % of questions were answered incorrectly for the table (*p* = 0.19). Out of 13 knowledge questions for the bronchiolitis topic, 14.2 % of questions were incorrectly answered for the harvest plot and 17.9 % of questions were incorrectly answered for the table (*p* = 0.22). Overall, the knowledge questions were answered correctly more often for the bronchiolitis topic (83.9 %) than the acute otitis media topic (74.3 %; *p* < 0.01). Participants answered significantly more efficacy questions correctly than safety questions (95.9 % efficacy; 81.0 % safety; *p* < 0.01). Among those who answered incorrectly, participants consistently chose the wrong intervention as more favorable in terms of safety. The survey knowledge questions and correct answers can be reviewed in Additional files 1 and 2.

## Discussion

The goal of this research was to explore the use of harvest plots, a novel form of data presentation [11], to promote the understanding of evidence from overviews of reviews. Standard tables and harvest plots were found to be rated equally, although neutrally, in terms of suitability and ease of understanding to summarize complex data from overviews of reviews. Harvest plots were found to be significantly more aesthetically pleasing. The proportion of correctly answered knowledge questions was similar for the harvest plots and tables. Given that the harvest plots were found to be similar to standard tables in terms of suitability and understanding, and aesthetically superior to standard tables, harvest plots are an equitable alternative for displaying the results of systematic reviews in overviews of reviews. However, given that both harvest plots and standard tables were rated neutrally in terms of suitability, understanding, and intuitiveness, neither format is ideal for the display of results from overviews of reviews.

There are several points to consider in developing and using harvest plots or other methods of data presentation. Of interest was the finding that neither harvest plots nor standard summary tables were ranked highly in terms of being easy to understand or intuitive. Further, overall there was a relatively high proportion of incorrectly answered questions suggesting inaccurate interpretation of results even among highly experienced researchers (median 3 systematic reviews and median 2 systematic reviews with meta-analyses published per respondent): 36.1 % and 28.9 % of questions were incorrectly answered for the acute otitis media and bronchiolitis topics, respectively. It is likely that if the general target audience of systematic reviews and overviews of reviews had been surveyed, they would have had an even greater proportion of incorrectly answered knowledge questions. Similarly, a previous statistical cognition experiment showed misinterpretation of forest plots (i.e., graphs representing the results of meta-analyses) with an average of 42 out of 63 questions answered correctly (67 %) among 279 researchers with experience in meta-analysis [18].

Consistent with previous research [19], we found some indication that respondents who were presented with a difference measure answered more knowledge questions correctly than those who were presented with a ratio measure suggesting that difference measures were understood better than ratio measures regardless of presentation format. Respondents consistently answered knowledge questions poorly for questions pertaining to adverse events. Importantly, respondents consistently reported the wrong direction of effect for adverse events, particularly when presented with a ratio measure. For example, for the acute otitis media topic when presented with the table, 78.6 % of respondents incorrectly answered false to the statement: “delayed antibiotics had significantly fewer adverse events with a NNT of 10”.

One possible explanation for the finding that neither of the two data presentation formats used in this study was intuitive or easy to understand is the large amount of information presented, including study information (number of studies, number of participants), effect estimates (using varied summary measures) and confidence intervals, measures of heterogeneity, numbers needed to treat, and ratings of the quality of evidence. During the development phase of the harvest plots, we were challenged with balancing the goals of providing key information for clinical decision-making while presenting the information in a way that could be readily understood and was not overwhelming for the reader. Further, an assumption we made when undertaking this study was that end users (particularly those with experience conducting systematic reviews) had a relatively strong understanding of data (and other concepts, e.g., statistical heterogeneity, GRADE assessments) typically presented in meta-analyses and systematic reviews. This study along with previous research [18] raises questions regarding the general understanding of statistical data and concepts from meta-analyses. Therefore, the volume of information combined with an inadequate understanding of the information presented may have influenced respondents’ perceptions of the data presentation formats. These findings highlight the need for further research regarding what information from knowledge syntheses (including overviews of reviews, systematic reviews, and meta-analyses) is most needed for clinical decision-making. Moreover, further research on how best to summarize and display this information will help inform knowledge translation strategies.

While this is one of few studies that has empirically evaluated the utility of different formats for presenting data from knowledge syntheses, it had several limitations. First, the response rate was low; therefore, results may not be widely generalizable. We assume that respondents may be those most interested in the topic and most knowledgeable about systematic reviews and meta-analyses; therefore, other end users of these knowledge synthesis products with less familiarity may find them even less intuitive, and may be therefore more likely to misinterpret results. A second limitation is that participants were given some guidance on how to interpret the harvest plots, so the results may overestimate ease of understanding. Thirdly, only two harvest plots and two standard tables, across two topics (acute otitis media and bronchiolitis) were assessed by survey respondents, which could also limit the generalizability of the findings. Finally, we created the harvest plots and tables based on a subset of outcomes from the original overviews of reviews. Applying this strategy to more outcomes, more comparisons, and other types of overviews may be more complicated with less ease of understanding and increased likelihood of misinterpretation.

## Conclusion

Neither harvest plots nor standard summary tables were ranked highly in terms of being easy to understand or intuitive, indicating that neither option is ideal for the graphical display of results from overviews of reviews. These results should be considered in knowledge translation efforts. Responses to the knowledge questions showed some misinterpretation of results of meta-analyses, even among systematic reviews with methodological expertise. The format of presentation appeared to have no added value in interpretation. Errors were more common for safety outcomes (i.e., knowing which intervention was preferable), with some indication of misinterpretation of relative (vs. absolute) measures. Reviewers should ensure that messages are clearly articulated and summarized in the text to avoid misinterpretation based on presentation of results.

## Additional files

Additional file 1: Survey for Acute Otitis Media. (PDF 63 kb) Additional file 2: Survey for Bronchiolitis. (PDF 64 kb)

## Abbreviations

CI: Confidence interval
NNT: Number needed to treat
IQR: Inter-quartile range
SD: Standard deviation
GRADE: Grading of Recommendations Assessment, Development, and Evaluation
