# Supplementary material for: An evaluation of harvest plots to display results of meta-analyses in overviews of reviews: a cross-sectional study
Source: BMC Med Res Methodol. 2015 Oct 26;15:91. doi: 10.1186/s12874-015-0084-0 (PMC4623293; doi:10.1186/s12874-015-0084-0)
Supplement: Additional file 1: — Survey for Acute Otitis Media. (PDF 63 kb) [file 12874_2015_84_MOESM1_ESM.pdf]

Harvest Plot Survey

Please complete the survey below.

Thank you!

Dear participant,

Thank you for taking the time to complete this survey. This project is an evaluation of different methods to display the results from systematic reviews.

This survey should take approximately 10 minutes of your time. The data will be used for research purposes only and your answers are completely anonymous.

Click on the Next button to navigate through this survey. Please make sure to read the figure notes carefully and answer all questions completely. This survey works optimally on a desktop computer; functionalities may not all appear as intended on other (e.g. mobile) devices. Do not use the Back button of your browser or return to previous pages once completed and please participate only once.

Should you wish to be notified of the publication results of this study, you may provide your e-mail address at the end of the survey. This information will be saved separately and cannot be associated with your responses to the survey.

At the end of the survey, you will also have the opportunity to enter your name in a draw for an iPad. You will have about a 1 in 200 chance of winning - good luck!

Thank you.

Lisa Hartling, PhD
Director, Cochrane Child Health Field
Associate Professor, Department of Pediatrics
University of Alberta

Harvest Plots for Acute Otitis Media

Explanation of Harvest Plots

These are harvest plots of six intervention comparisons for acute otitis media for two outcomes. The comparisons are: antibiotics vs. placebo, short course antibiotics vs. long course antibiotics, delayed antibiotics vs. immediate antibiotics, delayed antibiotics vs. none, decongestants/antihistamines vs. none, and topical analgesia vs. placebo.

The outcomes are pain and adverse events. Each row represents the outcomes for the specified comparison. Each 'plot' contains a bar showing the number of participants with the outcome for that comparison.
For example, the plot shows that:
a) among approximately 1000 participants, there was no difference between antibiotics compared with placebo for the outcome of pain at 24 hours;
b) among approximately 2500 participants, antibiotics were better than placebo for pain at 3-7 days, with number-needed-to-treat of 14; and
c) among approximately 2000 participants, there were fewer adverse events with placebo compared with antibiotics, with number-needed-to-treat of 7.

Bar color indicates quality of evidence (based on GRADE):
- green for high quality;
- yellow for moderate quality; and
- red for low quality.

[Inline Image: "aom_legend_10Dec2013.JPG"]

[Inline Image: "AOM_harvestplot_NNT fix.JPG"]

Please answer these questions with respect to the harvest plots above:

Q1 What were the results for antibiotics versus placebo (check all that apply)?

a) ☐ Antibiotics showed a small reduction in pain at 24 hours
b) ☑ Antibiotics significantly reduced pain at 3-7 days with a number needed to treat of 14
c) ☑ Antibiotics had significantly more adverse effects with a number needed to treat of 7
d) ☐ There were significantly more adverse events with placebo with a number needed to treat of 7

Q2 What were the results for short course versus long course antibiotics (check all that apply)?

a) ☐ There was no difference between short course and long course antibiotics for pain
b) ☑ There were no data comparing short course with long course antibiotics for pain
c) ☐ There were significantly more adverse events with short course versus long course antibiotics
d) ☑ There were significantly fewer adverse events with short course versus long course antibiotics

Q3 What were the results for delayed antibiotics (check all that apply)?

a) ☐ Delayed antibiotics showed a small but significant reduction in pain at 3-7 days
b) ☑ Delayed antibiotics had significantly fewer adverse events with a number needed to treat of 10
c) ☐ Delayed antibiotics had significantly more adverse events with a number needed to treat of 10
d) ☐ There were no data comparing adverse events between delayed antibiotics and no treatment

---

Q4 I have seen this type of graph before.

1 ☐ Yes
0 ☐ No

Q5 This type of graph is well-suited to summarize and graphically display results from meta-analysis.

0, not all all suited — 100, very much suited

(Place a mark on the scale above)

Q6 This type of graph is aesthetically pleasing.

0, not at all aesthetically pleasing — 100, very aesthetically pleasing

(Place a mark on the scale above)

Q7 This type of graph is easy to understand.

0, not at all easy to understand — 100, very easy to understand

(Place a mark on the scale above)

Q8 This type of graph is intuitive.

0, not at all intuitive — 100, very intuitive

(Place a mark on the scale above)

Table for Acute Otitis Media

Explanation of Table
This is a table of results for the same six intervention comparisons for acute otitis media.

[Inline Image: "AOM_table_10Dec2013 (2).JPG"]

Please answer these questions with respect to the table above:

Q9 What were the results for antibiotics versus placebo (check all that apply)?

- ☐ Antibiotics showed a small reduction in pain at 24 hours
- ☐ Antibiotics significantly reduced pain at 3-7 days with a number needed to treat of 14
- ☐ Antibiotics had significantly more adverse effects with a number needed to treat of 7
- ☐ There were significantly more adverse events with placebo with a number needed to treat of 7

Q10 What were the results for short course versus long course antibiotics (check all that apply)?

- ☐ There was no difference between short course and long course antibiotics for pain
- ☐ There were no data comparing short course with long course antibiotics for pain
- ☐ There were significantly more adverse events with short course versus long course antibiotics
- ☐ There were significantly fewer adverse events with short course versus long course antibiotics

Q11 What were the results for delayed antibiotics (check all that apply)?

- ☐ Delayed antibiotics showed a small but significant reduction in pain at 3-7 days
- ☐ Delayed antibiotics had significantly fewer adverse events with a number needed to treat of 10
- ☐ Delayed antibiotics had significantly more adverse events with a number needed to treat of 10
- ☐ There were no data comparing adverse events between delayed antibiotics and no treatment

---

Q12 I have seen this type of table before.

- ☐ Yes
- ☐ No

Q13 This type of table is well-suited to summarize and display results from meta-analysis.

0, not at all suited — 100, very much suited

(Place a mark on the scale above)

Q14 This type of table is aesthetically pleasing.

0, not at all aesthetically pleasing — 100, very aesthetically pleasing

(Place a mark on the scale above)

Q15 This type of table is easy to understand.

0, not at all easy to understand — 100, very easy to understand

(Place a mark on the scale above)

Q16 This type of table is intuitive.

0, not at all intuitive — 100, very intuitive

(Place a mark on the scale above)

Harvest Plots for Displaying Results from Meta-analyses

Q17 Were the harvest plots helpful to summarize the data from meta-analyses, in addition to the tables?

0, not helpful — 100, very helpful

(Place a mark on the scale above)

Q18 Could harvest plots be used in conjunction with tables to display results from meta-analyses?

- ☐ Yes
- ☐ Yes, but it could be improved
- ☐ No

Q19 Please describe how the harvest plots in conjunction with tables could be improved to best display results from meta-analyses.

____

Q20 Please describe why harvest plots in conjunction with tables could not be used to display results from meta-analyses.

____

Q21 Is there key information that is missing that would have been helpful in displaying the results from meta-analyses? Please describe in detail.

____

Personal Information

Please indicate your gender: Gender

☐ Female
☐ Male

Please indicate your academic degrees (select all that apply): Degrees

☐ BA/BSc or equivalent
☐ MA/MSc or equivalent
☐ MD or equivalent
☐ PhD or equivalent
☐ Other

Please specify other degrees: ______________________________

Please indicate the academic discipline in which the above qualifications was/were awarded: Eg. Medicine, Psychology

BA/BSc or equivalent: ______________________________

MA/MSc or equivalent: ______________________________

MD or equivalent: ______________________________

PhD or equivalent: ______________________________

Other: ______________________________

Please indicate your primary role/position: ______________________________
(Eg. Physician, Professor, Researcher, Research assistant, Other healthcare provider)

Please indicate the country of your academic affiliation:

☐ Australia
☐ Austria
☐ Canada
☐ China
☐ France
☐ Germany
☐ Italy
☐ New Zealand
☐ Portugal
☐ Spain
☐ UK/Ireland
☐ USA
☐ Other

(If you are affiliated with institutions in several countries, please refer to the one you consider the most important)

Please specify other country of affiliation: ______________________________

____

Please indicate the number of (if none, enter zero):

1 Systematic reviews you have published (please provide an estimate if you cannot recall): ______________________________

Please indicate the number of systematic reviews you have published that contain at least one meta-analysis: ______________________________

2 Journal articles on development of methods for systematic reviews that you have published: ______________________________

3 Journal articles specifically on development of meta-analytical methods you have published: ______________________________

4 Other texts relevant to meta-analysis (eg. book chapters, letters, editorials) you have published: ______________________________

If you would like to receive a summary of the results of this study, please leave your email address.

Your contact data will be saved separately and cannot be associated with your response to the survey.

I would like to be contacted about the study results.
- ☐ Yes
- ☐ No

Email Address: ________________________________

If you would like to be entered into a draw for a chance to win an iPad, please leave your email address. ________________________________
