# Supplementary material for: An evaluation of harvest plots to display results of meta-analyses in overviews of reviews: a cross-sectional study
Source: BMC Med Res Methodol. 2015 Oct 26;15:91. doi: 10.1186/s12874-015-0084-0 (PMC4623293; doi:10.1186/s12874-015-0084-0)
Supplement: Additional file 2: — Survey for Bronchiolitis. (PDF 64 kb) [file 12874_2015_84_MOESM2_ESM.pdf]

Harvest Plot Survey

Please complete the survey below.

Thank you!

Harvest Plot Survey

Dear participant,

Thank you for taking the time to complete this survey. This project is an evaluation of different methods to display the results from systematic reviews.

This survey should take approximately 10 minutes of your time. The data will be used for research purposes only and your answers are completely anonymous.

Click on the Next button to navigate through this survey. Please make sure to read the figure notes carefully and answer all questions completely. This survey works optimally on a desktop computer; functionalities may not all appear as intended on other (e.g. mobile) devices. Do not use the Back button of your browser or return to previous pages once they are completed, and please participate only once.

Should you wish to be notified of the publication results of this study, you may provide your e-mail address at the end of the survey. This information will be saved separately and cannot be associated with your responses to the survey.

At the end of the survey, you will also have the opportunity to enter your name in a draw for an iPad. You will have about a 1 in 200 chance of winning - good luck!

Thank you.

Lisa Hartling, PhD
Director, Cochrane Child Health Field
Associate Professor, Department of Pediatrics
University of Alberta

Harvest Plots for Bronchiolitis

Explanation of Harvest Plots

These are harvest plots of six intervention comparisons for bronchiolitis for two outcomes. The comparisons are: glucocorticoid vs. placebo, bronchodilator vs. placebo, epinephrine vs. placebo, epinephrine vs. bronchodilator, 3% hypertonic saline vs. 0.9% saline, and chest physiotherapy vs. standard care.

The outcomes are clinical score and length of stay. Each row represents the outcomes for the specified comparison.
Each 'plot' contains a bar showing the number of participants with the outcome for that comparison.
For example, the plot shows that:
a) among approximately 350 participants, there was no difference between bronchodilator compared with placebo on length of stay;
b) among over 200 participants, there was no difference between epinephrine compared with placebo on clinical score or length of stay; and
c) among approximately 100 participants, chest physiotherapy was better than standard care at reducing clinical score at 1-3 days.

Bar color indicates quality of evidence (based on GRADE):
- green for high quality;
- yellow for moderate quality; and
- red for low quality.

[Inline Image: "bronchiolitis_legend_10Dec2013.JPG"]

[Inline Image: "bronchiolitis_harvestplot_10Dec2013.JPG"]

Please answer these questions with respect to the harvest plots above:

What were the results for glucocorticoids vs. placebo (check all that apply):

Q1

- [ ] There was a significantly longer length of stay with glucocorticoids compared with placebo
- [x] There was no difference in clinical score at 1 to 3 days
- [ ] There was a small difference in clinical score at 1 to 3 days
- [x] There was no significant difference in length of stay between glucocorticoids and placebo

What were the results for epinephrine vs. other bronchodilators (check all that apply):

Q2

- [x] There was a significant benefit for epinephrine compared with other bronchodilators in terms of clinical score at 60 minutes and 120 minutes
- [ ] There was a significant benefit for other bronchodilators compared with epinephrine in terms of clinical score at 60 minutes and 120 minutes
- [ ] There was a significantly greater length of stay with epinephrine compared with other bronchodilators
- [x] There was a significantly greater length of stay with other bronchodilators compared with epinephrine

What were the results for 3% hypertonic saline vs. 0.9% saline (check all that apply):

Q3

- [ ] There was a significant difference with improved clinical score at 1-3 days for 0.9% saline
- [x] There was a significant difference with improved clinical score at 1-3 days for 3% hypertonic saline
- [ ] There was a significantly longer stay with 3% hypertonic saline
- [x] There was a significantly longer stay for 0.9% saline
- [ ] There was not enough evidence to compare 3% hypertonic saline with 0.9% hypertonic saline for length of stay

---

I have seen this type of graph before. Q4

- [ ] Yes
- [ ] No

This type of graph is well-suited to summarize and graphically display results from meta-analysis. Q5

0, not all all suited — 100, very much suited

(Place a mark on the scale above)

This type of graph is aesthetically pleasing. Q6

0, not at all aesthetically pleasing — 100, very aesthetically pleasing

(Place a mark on the scale above)

This type of graph is easy to understand. Q7

0, not at all easy to understand — 100, very easy to understand

(Place a mark on the scale above)

This type of graph is intuitive. Q8

0, not at all intuitive — 100, very intuitive

(Place a mark on the scale above)

Table for Bronchiolitis

Explanation of Table
This is a table of results for the same six intervention comparisons for bronchiolitis.

[Inline Image: "bronchiolitis_table_10Dec2013_no gridlines.JPG"]

Please answer these questions with respect to the table above:

What were the results for glucocorticoids vs. placebo (check all that apply): Q9

- ☐ There was a significantly longer length of stay with glucocorticoids compared with placebo
- ☐ There was no difference in clinical score at 1 to 3 days
- ☐ There was a small difference in clinical score at 1 to 3 days
- ☐ There was no significant difference in length of stay between glucocorticoids and placebo

What were the results for epinephrine vs. other bronchodilators (check all that apply): Q10

- ☐ There was a significant benefit for epinephrine compared with other bronchodilators in terms of clinical score at 60 minutes and 120 minutes
- ☐ There was a significant benefit for other bronchodilators compared with epinephrine in terms of clinical score at 60 minutes and 120 minutes
- ☐ There was a significantly greater length of stay with epinephrine compared with other bronchodilators
- ☐ There was a significantly greater length of stay with other bronchodilators compared with epinephrine

What were the results for 3% hypertonic saline vs. 0.9% saline (check all that apply): Q11

- ☐ There was a significant difference with improved clinical score at 1-3 days for 0.9% saline
- ☐ There was a significant difference with improved clinical score at 1-3 days for 3% hypertonic saline
- ☐ There was a significantly longer stay with 3% hypertonic saline
- ☐ There was a significantly longer stay 0.9% saline
- ☐ There was not enough evidence to compare 3% hypertonic saline with 0.9% hypertonic saline for length of stay

---

I have seen this type of table before. Q12

- ☐ Yes
- ☐ No

This type of table is well-suited to summarize and display results from meta-analysis. Q13

0, not at all suited — 100, very much suited

(Place a mark on the scale above)

This type of table is aesthetically pleasing. Q14

0, not at all aesthetically pleasing — 100, very aesthetically pleasing

(Place a mark on the scale above)

This type of table is easy to understand. Q15

0, not at all easy to understand — 100, very easy to understand

(Place a mark on the scale above)

This type of table is intuitive. Q16

0, not at all intuitive — 100, very intuitive

(Place a mark on the scale above)

Harvest Plots for Displaying Results from Meta-analyses

Were the harvest plots helpful to summarize the data from meta-analyses, in addition to the tables? Q17

0, not helpful — 100, very helpful

(Place a mark on the scale above)

Could harvest plots be used in conjunction with tables to display results from meta-analyses? Q18

☐ Yes
☐ Yes, but it could be improved
☐ No

Please describe how the harvest plots in conjunction with tables could be improved to best display results from meta-analyses. Q19

____

Please describe why harvest plots in conjunction with tables could not be used to display results from meta-analyses. Q20

____

Is there key information that is missing that would have been helpful in displaying the results from meta-analyses? Please describe in detail. Q21

____

Personal Information

Please indicate your gender: Q22
- ☐ Female
- ☐ Male

Please indicate your academic degrees (select all that apply): Q23
- ☐ BA/BSc or equivalent
- ☐ MA/MSc or equivalent
- ☐ MD or equivalent
- ☐ PhD or equivalent
- ☐ Other

Please specify other degrees: ______________________

Please indicate the academic discipline in which the above qualifications was/were awarded:
Eg. Medicine, Psychology

BA/BSc or equivalent: Q24 ______________________

MA/MSc or equivalent: ______________________

MD or equivalent: ______________________

PhD or equivalent: ______________________

Other: ______________________

Please indicate your primary role/position: Q25 ______________________
(Eg. Physician, Professor, Researcher, Research assistant, Other healthcare provider)

Please indicate the country of your academic affiliation: Q26
- ☐ Australia
- ☐ Austria
- ☐ Canada
- ☐ China
- ☐ France
- ☐ Germany
- ☐ Italy
- ☐ New Zealand
- ☐ Portugal
- ☐ Spain
- ☐ UK/Ireland
- ☐ USA
- ☐ Other

(If you are affiliated with institutions in several countries, please refer to the one you consider the most important)

Please specify other country of affiliation: Q27 ______________________

____

Please indicate the number of (if none, enter zero):

1 Systematic reviews you have published (please provide an estimate if you cannot recall): Q28 ______________________

Please indicate the number of systematic reviews you have published that contain at least one meta-analysis: Q29 ______________________

2 Journal articles on development of methods for systematic reviews that you have published: Q30 ______________________

3 Journal articles specifically on development of meta-analytical methods you have published: Q31 ______________________

4 Other texts relevant to meta-analysis (eg. book chapters, letters, editorials) you have published: Q32 ________________________________

If you would like to receive a summary of the results of this study, please leave your email address.

Your contact data will be saved separately and cannot be associated with your response to the survey.

I would like to be contacted about the study results.	☐ Yes<br>☐ No
Email Address:	__________________
If you would like to be entered into a draw for a chance to win an iPad, please leave your email address.	__________________
